# Insights on Guerbet Reaction: Production of Biobutanol From Bioethanol Over a Mg–Al Spinel Catalyst

**DOI:** 10.3389/fchem.2022.945596

**Published:** 2022-07-14

**Authors:** M. A. Portillo Crespo, F. Vidal-Barrero, Lola Azancot, Tomas Ramírez Reina, M. Campoy

**Affiliations:** ^1^ Departamento de Ingeniería Química y Ambiental, Escuela Técnica Superior de Ingeniería, Universidad de Sevilla, Sevilla, Spain; ^2^ Department of Inorganic Chemistry and Material Sciences Institute of Seville, Universidad de Sevilla-CSIC, Sevilla, Spain; ^3^ Department of Chemical and Process Engineering, University of Surrey, Guildford, United Kingdom

**Keywords:** n-butanol, green fuels, green chemicals, renewable sources, sustainable catalysis

## Abstract

The production of biobutanol from bioethanol by the Guerbet reaction is an alternative pathway to renewable sources. The commercial viability of this green route requires improvements in the process development. This study experimentally examines the influence of operating conditions on the performance of a Mg–Al spinel catalyst prepared from hydrotalcite precursors. This catalyst demonstrates an exceptional performance in the Guerbet reaction with a promising activity/butanol selectivity balance, excellent long-term stability, and very-low-carbon footprint (CO_2_ generation as by-products is minimal). This study showcases a systematic strategy to optimize the reaction parameters in the Guerbet reaction for biobutanol production using an advanced spinel catalyst. Upon carefully adjusting temperature, pressure, space velocity, and reactants co-feeding, very promising conversion (35%) and butanol selectivity values (48%) were obtained.

## Graphical Abstract

Heterogeneous catalysis will play a major role to enable the production of green fuels and chemicals reducing the carbon footprint.
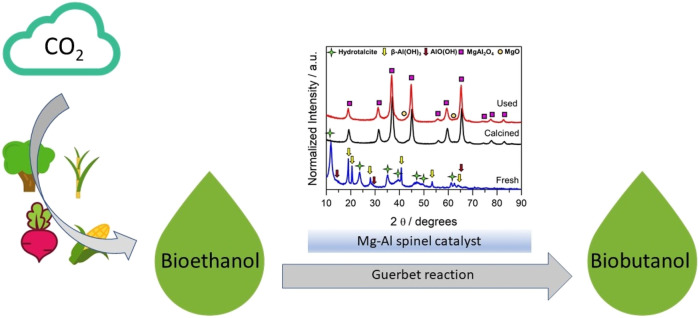

## Introduction

n-Butanol is a potential engine fuel owing to its properties closely resembling those of gasoline (Le Van Mao et al., 1989; Iglesia et al., 1997; Riittonen et al., 2012), and it is also an important solvent and intermediate chemical to produce paints, coatings, adhesives, and plasticizers (Uyttebroek et al., 2015). Nowadays, most n-butanol is synthesized through the petrochemical pathway from propylene and syngas, where in a first stage, propylene is hydroformylated to butyraldehyde (oxo process), and then, in a second stage, it is further hydrogenated to n-butanol (Gehrmann and Tenhumberg, 2020). Before the petrochemical route, between 1900 and 1960, commercial production of n-butanol was dominated by ABE fermentation, which made use of sugars from cereal grains or molasses as raw material. The increase in the price of these substrates and the low oil prices made ABE fermentation not cost-competitive against the petrochemical pathway, and many plants were closed (Ndaba et al., 2015). The need for “green technologies” to solve global warming problems in the 21st century has fostered interest in sustainable processes for production of n-biobutanol. An alternative sustainable and environmentally friendly route to produce n-biobutanol is by catalytic synthesis from bioethanol through the Guerbet reaction (Dvornikoff and Farrar, 1957) (Supplementary Section S1 of Supporting Information). This route is of special interest owing to the large availability of bioethanol in the market.

An efficient catalyst must achieve high ethanol conversion and selectivity to butanol, considering that there are a thermodynamically unfavorable dehydrogenation of ethanol and the formation of side-products because of the uncontrolled base-catalyzed aldol condensation of highly reactive acetaldehyde (Zhang et al., 2016). An excellent review on homogeneous and heterogeneous catalysts for the Guerbet reaction was reported by Gabriëls et al. (2015) and Sun and Barta (2018), while an update on catalysts design for n-butanol synthesis from ethanol is provided by Zhang et al. (2016). Homogeneous catalysts typically feature a precious metal for the sequential dehydrogenation/hydrogenation steps and an inorganic base to promote the aldol condensation step (Zhang et al., 2016). The metal dehydrogenates the alcohol to a carbonyl intermediate by the formation of a metal hydride in the first reaction (R1), which then transfers hydrogen to the aldol addition product (R4). When a transition metal is implemented, it favors dehydrogenation step at a lower reaction temperature (150–250°C) owing to the lower activation energy. High butanol selectivity from ethanol has been achieved with homogeneous catalysts, such as ruthenium-based systems with selectivity higher than 90% and ethanol conversion up to 31% (Dowson et al., 2013; Wingad et al., 2015), and bifunctional iridium catalyst coupled with bulky nickel or copper hydroxides with selectivity higher than 99% and ethanol conversion up to 37% (Chakraborty et al., 2015). Despite the high selectivity to n-butanol, the space–time yield achieved with homogeneous catalysts is still far away for commercial application, and the development of more active catalysts is needed (Zhang et al., 2016). In addition, the use of a homogeneous base poses some practical problems, such as reactor vessel corrosion and product–catalyst separation (Gabriëls et al., 2015).

Heterogeneous catalysts for the Guerbet reaction have been reviewed by Gabriels et al. (2015) and Kozlowski and Davis (2013). Reported butanol selectivity from ethanol with heterogeneous catalysts is lower than that with homogeneous catalysts (Zhang et al., 2016). Mg–Al mixed oxide catalysts derived from hydrotalcite precursors have received great attention as very promising catalysts for the Guerbet condensation because of the acid-basic properties, high surface area, structural stability at high temperatures, absence of toxic metals, and competitive cost (Larina et al., 2019). A palladium-doped Mg/Al mixed oxide derived from hydrotalcyte calcination (0.5%Pd-HT; at 200°C, 30 bar) (Arjona Antolín, 2014) achieved 80% n-butanol selectivity and 17% ethanol conversion after 17 h of reaction time in operation in liquid phase. Likewise to this, other authors (Marcu et al., 2013) studied the effect of the different metals (Pd, Ag, Mn, Fe, Cu, Sm, and Yb) over Mg–Al mixed oxide. That study showed that palladium-doped MgAl provided a high selectivity (70%) at 200°C. Authors stated that Pd nanoparticles were the optimum hydrogenation–dehydrogenation component of a bifuncional catalyst. In the case of copper (Cu/MgAl), the strong basic sites are active in butanol synthesis, while the acid sites mostly catalyze ethanol dehydration to ethylene and diethyl ether. Larina et al. (2019) used a Mg/Al catalyst with a mole ratio of Mg/Al = 2 in gas phase, obtaining 26.9% ethanol conversion and 65.2% butanol selectivity. In recent times, the same authors used a Mg–Al–Ce hydrotalcites with Mg/(Al + Ce) = 2 and obtained a slightly higher butanol selectivity (68%) (Larina et al., 2021). Perrone et al. (2021) used a Cu–MgAl (O) catalyst at 280°C, obtaining a selectivity to butanol and hexanol of 62 and 10%, respectively. The addition of La (CuLa–MgAl (O)) increased the number of basic sites compared to that of Cu–MgAl (O), which changed the selectivity to butanol (16.7%) and 1-hexanol (41.7%) at 280°C. The addition of lanthanum and consequently the increase in the catalyst basicity was related to the increasing of long-chain alcohols as a result of the enolate formation during the aldol condensation of Guerbet coupling.

In order to make this route commercially viable (the Guerbet condensation reaction of ethanol to mainly butanol), improvements in catalyst design and process development need to be achieved. In recent years, scientific publications have appeared using hydrotalcite materials as catalysts (or catalyst precursors) in the alcohol condensation reactions, in both batch systems and fixed bed continuous reactors (Arjona Antolín, 2014). All these publications are focused on improving the performance of the catalysts, but there is a scarcity of works on practical aspects for their industrial application, including, for instance, optimization of operating conditions, catalyst stability, or product recycling. Therefore, a detailed study of operating conditions is deemed necessary to fill this literature gap and shed some light on the optimization of the Guerbet reaction toward biobutanol synthesis. In addition, long-term tests are necessary to validate industrial operations. In the literature, Xi et al. (2020) carried out a catalytic test which lasted 160 h, but most of the published articles performed short-term trials. For instance, Cimino et al. (2018) tested a Ru/MgO catalyst for only 6 h. Under this scenario, the aim of this work is to study the effect of operating conditions in the performance of a Mg–Al spinel catalyst prepared from hydrotalcite precursors, for the direct synthesis of butanol from ethanol. The catalyst was thoroughly characterized pre- and post-reaction using nitrogen adsorption–desorption isotherm, X-ray diffraction (XRD), NH_3_/CO_2_ temperature-programmed desorption (NH_3_/CO_2_-TPD), and thermogravimetric analysis (TGA). The effect of key reaction parameters such as temperature, pressure, liquid hourly space velocity (LHSV), and catalyst stability were analyzed. Hydrogen availability to promote the aldol product hydrogenation targeting final alcohol is of paramount importance, and our work genuinely described the impact of hydrogen co-feeding in the overall process.

## Materials and Methods

### Experimental Facility Setup and Catalytic Tests

Supplementary Section S1 of Supporting Information shows the catalytic reactor experimental setup. The calcined mixed oxide spinel catalyst (MgAl_2_O_4_) was not pretreated. It was stabilized by feeding ethanol at 300°C, atmospheric pressure (0.39 bar of ethanol partial pressure), and LHSV = 0.235 h^−1^. The total flow of ethanol fed was 0.0106 ml/min to obtain an LHSV = 0.235 h^−1^, since the catalyst volume is 2.7 ml. In tests at different LHSV values, the total flow of ethanol fed can be easily calculated, taking into account that the catalyst volume is the one mentioned above.

Once the catalyst was stabilized, all the tests were carried out without changing the catalyst sample. Experimental data were obtained operating continuously by averaging the product reaction values of each experiment with a minimum of 10 chromatographic analyses (each one every 38 min).

The ethanol conversion, the product distribution, and the butanol productivity were respectively calculated as follows:

$$\text{Conversion},\text{XEtOH}\left(\%\right)=\frac{\text{C}\;\text{mol}\;\text{of}\;\text{ethanol}\;\text{fed}-\text{C}\;\text{mol}\;\text{of}\;\text{ethanol}\;\text{at}\;\text{reactor}\;\text{outlet}\;}{\text{C}\;\text{mol}\;\text{of}\;\text{ethanol}\;\text{fed}}\cdot 100$$

$$\text{Carbon}\;\text{product}\;\text{selectivity}\left(\%\right)=\frac{\text{C}\;\text{mol}\;\text{of}\;\text{product}\;\text{i}}{\text{C}\;\text{mol}\;\text{of}\;\text{total}\;\text{products}\;}\cdot 100$$

$$\text{Butanol}\;\text{productivity},\text{PButOH}\left(\frac{\text{g}}{{\text{kg}}_{\text{cat}\cdot }\text{h}}\right)=\frac{{\text{Butanol}}_{\text{out}}\left(\frac{\text{g}}{\text{h}}\right)}{\text{catalyst}\;\text{weight}\left(kg\right)}$$

### Catalyst Preparation and Characterization

The magnesium–aluminum hydrotalcite precursor was used to obtain a mixed oxide MgAl_2_O_4_ catalyst by thermal decomposition. The appropriate ratio of magnesium/aluminum was used in the precursor synthesis to obtain a stoichiometric spinel (MgAl_2_O_4_) based on the phase diagram (72 wt% Al_2_O_3_ and 28 wt% MgO) (Braulio et al., 2011). The hydrotalcite was prepared by co-precipitation method using an aqueous solution of 2 M NaOH and 0.5 M Na_2_CO_3_, as a precipitation agent. The synthesis took place in an automatically controlled titration system. The solution was added in a constant drip into an aqueous solution of metal nitrate salts, Mg(NO_3_)_2_·6H_2_O and Al (NO_3_)_3_·9H_2_O (molar ratio Mg^2+^/Al^3+^ = 0.5), and the mixture was maintained under continuous stirring at 70°C until reaching pH 10. The final solution was aged 18 h at the same temperature in stirring. After that, the precipitate solution was filtrated and washed with deionized water. Nitrates and carbonates were removed during the calcination process at high temperatures. The filter cake obtained was dried at 100°C overnight and finally calcined at 900°C (10°C/min) for 24 h, thus obtaining the calcined MgAl_2_O_4_ mixed oxide catalyst. The Mg/Al ratio is selected to achieve an adequate balance acid-basic sites as showcased in the acidity studies *vide infra* in the Results section. The use of Mg–Al spinel catalyst brings some novelty since the spinel structure is scarcely studied in this reaction compared to standard mixed oxide systems.

Nitrogen adsorption–desorption isotherm was used to analyze the textural properties of the calcined catalyst. The isotherm was realized at liquid nitrogen temperature in a Micromeritic Tristan II apparatus. First, the sample was degassed at 250°C for 4 h in a vacuum. The surface area was determined using the Brunauer–Emmett–Teller (BET) method, and the pore size was calculated using the Barret–Joyner–Halenda (BJH) method.

XRD was conducted to identify the crystalline structure and phases present in the fresh (hydrotalcite precursor), calcined, and spent catalysts. The measurement was carried out on a Siemens D-500 diffractometer using a Ni-filtered Cu kα radiation (40 mA and 45 kV), from 10º to 90° 2θ (0.05° step size and 300 s per step).

NH_3_-TPD and CO_2_-TPD were performed in homemade equipment using a U-shape quartz tube with 100 mg of sample. In both cases, the samples were pretreated in an inert gas (50 ml/min He) until 200°C for 1 h to remove the water physisorbed and then were cooled down to 50°C. Afterward, 30 ml/min of CO_2_ or NH_3_/He (5 vol%) was passed through the sample until saturation (30 min). Then the samples were purged with He (30 ml/min) for 1 h. In addition, the temperature was increased from 50 to 900°C in He (30 ml/min) with 10°C/min heating ramp. The effluent gases from the CO_2_ or NH_3_ desorption were monitored by mass spectrometry (MS PFEIFFER Vacuum Prisma Plus controlled by QUadera^®^ software). The mass/charge ratios followed in NH_3_-TPD were m/z = 16, 17, and 18. Owing the presence of water signals (m/z = 18, 17) in the gas flow, it is necessary to make a signal correction for an accurate ammonia formation profile to deduct m/z = 17 contribution of water. The difference between the total signal and the water m/z = 18, representing 26% of the signal, was considered as a correction factor. For CO_2_-TPD, the CO_2_ (m/z = 44) signal was monitored.

Carbon deposition and thermal stability were studied by TGA on a TA Instrument SDT Q600 equipment. The experiment covered a range of temperatures from RT to 900°C (rate 10°C/min) under airflow (100 ml/min).

## Results and Discussion

### Catalyst’s Characterization

**FIGURE 1:**
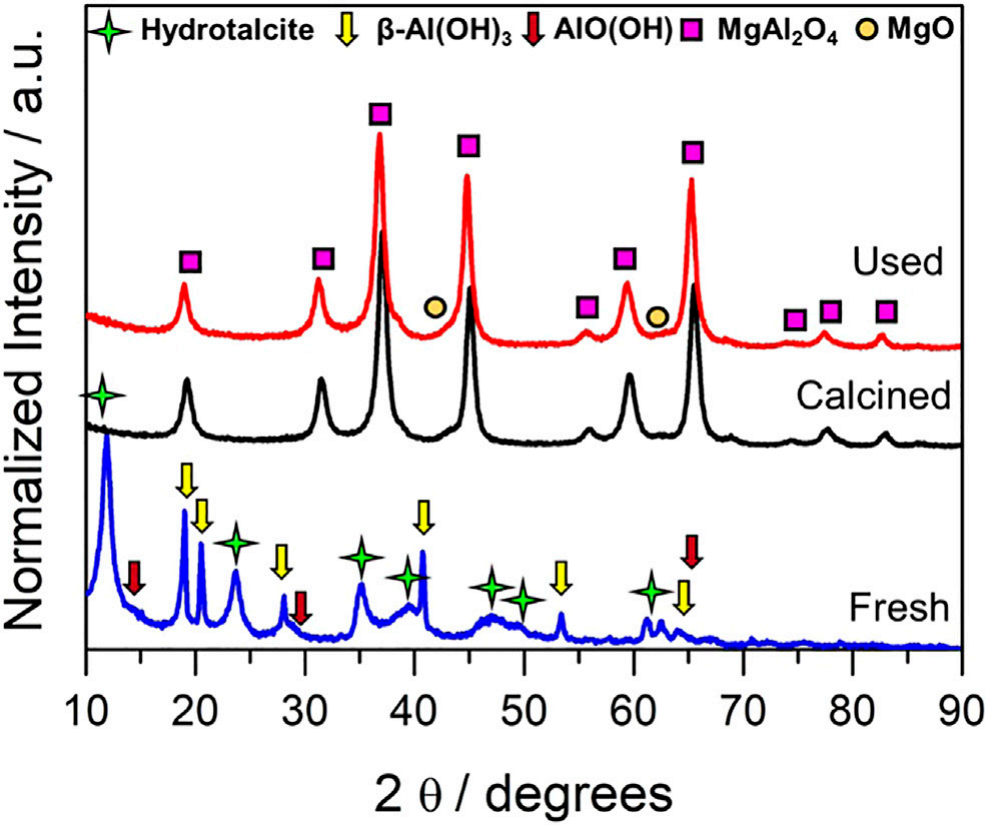
XRD pattern of hydrotalcite precursor (fresh), calcined, and spent mixed oxide catalysts. Note: a.u. is equivalent to arbitrary units.

Textural properties are presented in Supplementary Section S3 of Supporting Information, showcasing the mesoporous nature of the synthesized catalyst. As for the structural analysis, Figure 1 presents the XRD pattern of the fresh catalyst (referred to as hydrotalcite precursor) and the calcined and the spent mixed oxide catalyst (MgAl_2_O_4_). As shown in Figure 1, the hydrotalcite precursor obtained using co-precipitation methods presents a mixture of different phases. The formation of some side phases apart from a pure hydrotalcite structure was associated with the ratio of M^2+^/M^3+^, as suggested by Cavani et al. (1991) in a very early study. It was identified that the reflection planes (003), (006), and (009) of the layered hydrotalcite structure at 2θ 11.6, 23.6, and 35° corresponding with the 3R rhombohedral plane’s reflection ascribed to the magnesium/aluminum hydrotalcite (MgAl_2_CO_3_(OH)_16_·4H_2_O) (Pérez-Ramírez et al., 2007). Together with hydrotalcite diffractions, β-Al(OH)_3_ (bayerite) and AlO(OH) (boehmite) diffraction peaks are observed, and the presence of Mg(OH)_2_ phase cannot be discarded owing to overlapping with aluminum species (Dębek et al., 2016; Lee et al., 2016). These species are considered promoters of the double-layer hydrotalcite compound in which the Al and Mg ions are integrated into the layered structure, giving rise to the hydrotalcite formation (Lee et al., 2016). This fact suggested that the hydrotalcite phase is not formed almost completely or it is defective, which could affect the structure of the mixed oxide. The mixed oxide pattern (MgAl_2_O_4_), obtained after the hydrotalcite calcination at 900°C, presents crystalline and well-defined peaks of the plane reflections (111), (220), (311), (222), (400), (422), (511), and (440) ascribed to magnesium aluminate spinel. Furthermore, we identify small reflections of the MgO phase at = 42.97 and 62.29°. The presence of a MgO phase out of the spinel structure could be associated to the aluminum species observed in the hydrotalcite compound, generating a non-stoichiometric mixed oxide. Further evaluation of the XRD analysis as suggested by Carvalho et al. (2012) would be beneficial. Nevertheless, this is beyond the objectives of this work whose main aim is to cover broad parametric studies of the reaction conditions for a fine catalyst.

For the sake of crystalline structure stability analysis during the reaction, the spent catalyst was also studied. As depicted in Figure 1, there are no significant changes in the X-ray pattern of the sample whose dominant phases remain as MgAl_2_O_4_ and MgO in fair agreement with its calcined unreacted counterpart. A slight shift, though, to lower 2θ angles was appreciated in comparison to the calcined sample. This could indicate a possible inversion in the spinel, where the Mg^2+^ and Al^3+^ exchange positions from the tetrahedral and octahedral sites. It is important to highlight that we did not observe diffractions ascribed to carbon phases after the reaction. Hence, no crystalline carbon is formed, indicating that catalyst deactivation by carbon poisoning might not be an issue in this material under the studied reaction conditions.

**FIGURE 2:**
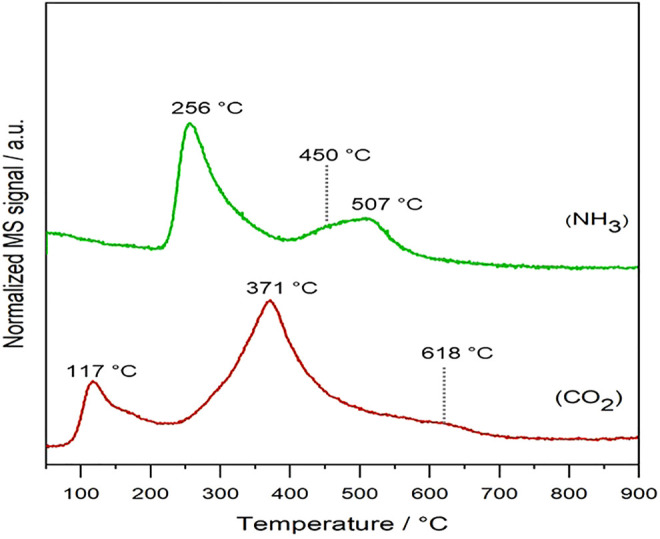
NH_3_-TPD and CO_2_-TPD patterns of the MgAl_2_O_4_ catalyst. Note: a.u. is equivalent to arbitrary units.

The surface acid–base properties of the mixed oxide catalyst (MgAl_2_O_4_) were evaluated by NH_3_-TPD and CO_2_-TPD. Both profiles are shown in Figure 2. NH_3_-TPD was carried out to evaluate the strength of acid sites in the catalyst surface. We differentiate two zones associated with the acid strength, depending on the NH_3_ temperatures desorption: moderate (200–400°C) and strong (400–800°C) (Madduluri et al., 2020). The peak at a lower temperature, 256°C, is associated with reversible adsorption H-bonded ascribed to hydroxyl groups presence over the surface, considered as BrØnsted acid sites (Song et al., 2021). Alternatively, the peaks at high temperatures (450–510°C) are related to Lewis acid sites, showcasing the interaction with accessible Al^+3^ cations in Al^3+^–O^2−^–Mg^2+^, because of possible defects present over the spinel surface (He et al., 2015). Furthermore, Coleman et al. (2009) associated the Lewis acid sites with the presence of amorphous AlO_*y*_ species that provoke an electron-deficient Al^3+^.

CO_2_-TPD was performed to study the surface basicity in the mixed oxide (Figure 2). The catalyst exhibits low–moderate basic sites at lower temperatures mainly, although it is possible to distinguish weak desorption at 618°C, indicating strong basic sites formation. Song et al. (2021) ascribe these peaks to the formation of the different carbonate geometries because of reactive CO_2_ absorption. The peak at 180°C could be related to the bicarbonate anions associated with the presence of hydroxyl groups; meanwhile, the peak at 371°C is ascribed to bidentate carbonates on acid–base sites (Al^3+^–O^2−^ or Mg^2+^–O^2−^) (Díez et al., 2003). Furthermore, formation of monodentate carbonates with strong basicity (high temperature) associated with the presence of pure MgO phase outside the spinel structure cannot be disregarded. Overall, our catalyst presents both medium and strong acidic sites, with medium strength sites being the dominant ones.

### Effects of Reaction Conditions

#### Carbon Deposits

**FIGURE 3:**
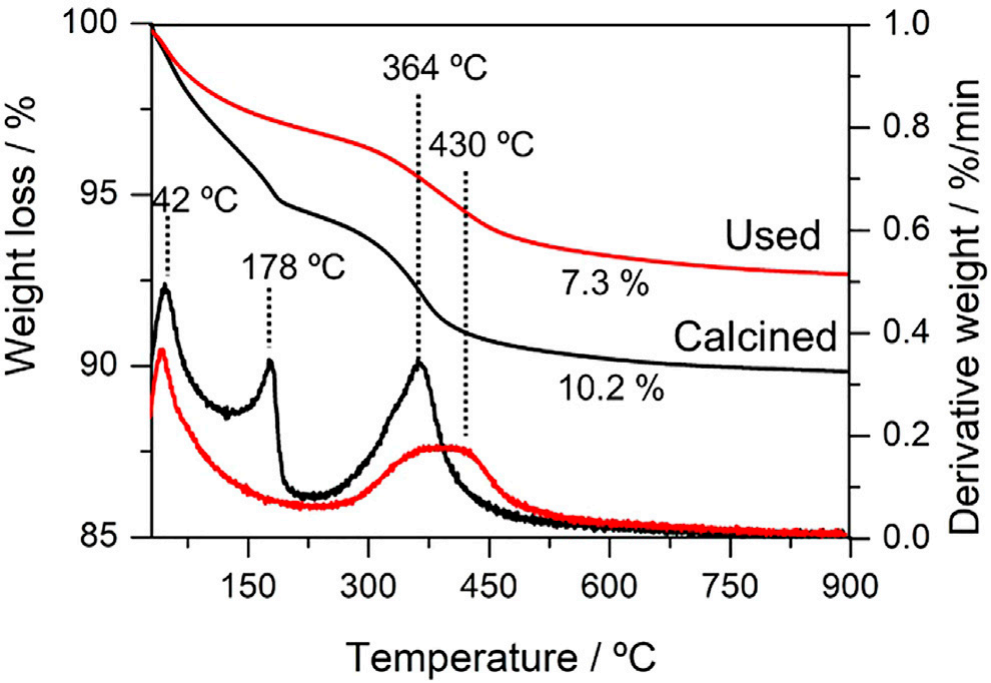
TGA profiles of calcined and spent catalysts (MgAl_2_O_4_).

Formation of carbon deposits and their nature in the spent catalyst was analyzed by TGA. Figure 3 shows the weight loss curves and their derivatives of the used catalyst. In addition, the calcined catalyst was tested as a reference to identify the possible weight loss not ascribed to carbon deposition since no crystalline carbonaceous species are intended from the XRD analysis. The TGA showed different weight loss ascribed to oxidation processes at different temperatures. The first process at 42°C common in both samples is associated with physically absorbed water on the catalyst’s surface (Barbosa et al., 2018). The second weight loss event at 178°C only appears in the calcined sample and may be because of the dehydration process of the adsorbed water in the spinel structure (Barbosa et al., 2018). Considering the previous oxidation processes, the weight loss at 364°C observed in the MgAl_2_O_4_ calcined sample could be associated with an external dihydroxylation on the surface of the material because of hydration of the material or a decarbonization process on the surface (Barbosa et al., 2018). Furthermore, another oxidation process in the same temperature range for the spent catalysts overlaps with another oxidation event at 430°C. Sahoo et al. (2003) reported that coke species oxidized in the temperature range 350–450°C are surface species classified as amorphous-type carbon (Al–Swai et al., 2021). Regarding the weight loss percentage, the calcined sample presents the highest weight loss because of a high concentration of water in the structure. Overall, the TGA suggests no major carbon deposits in the catalysts, in good agreement with the XRD data and indicating that the developed catalysts are robust and structurally stable for the Guerbet reaction. Hence, in the next sections, we will discuss the reaction condition optimization for this promising catalytic formulation.

#### Catalyst Stability

**FIGURE 4:**
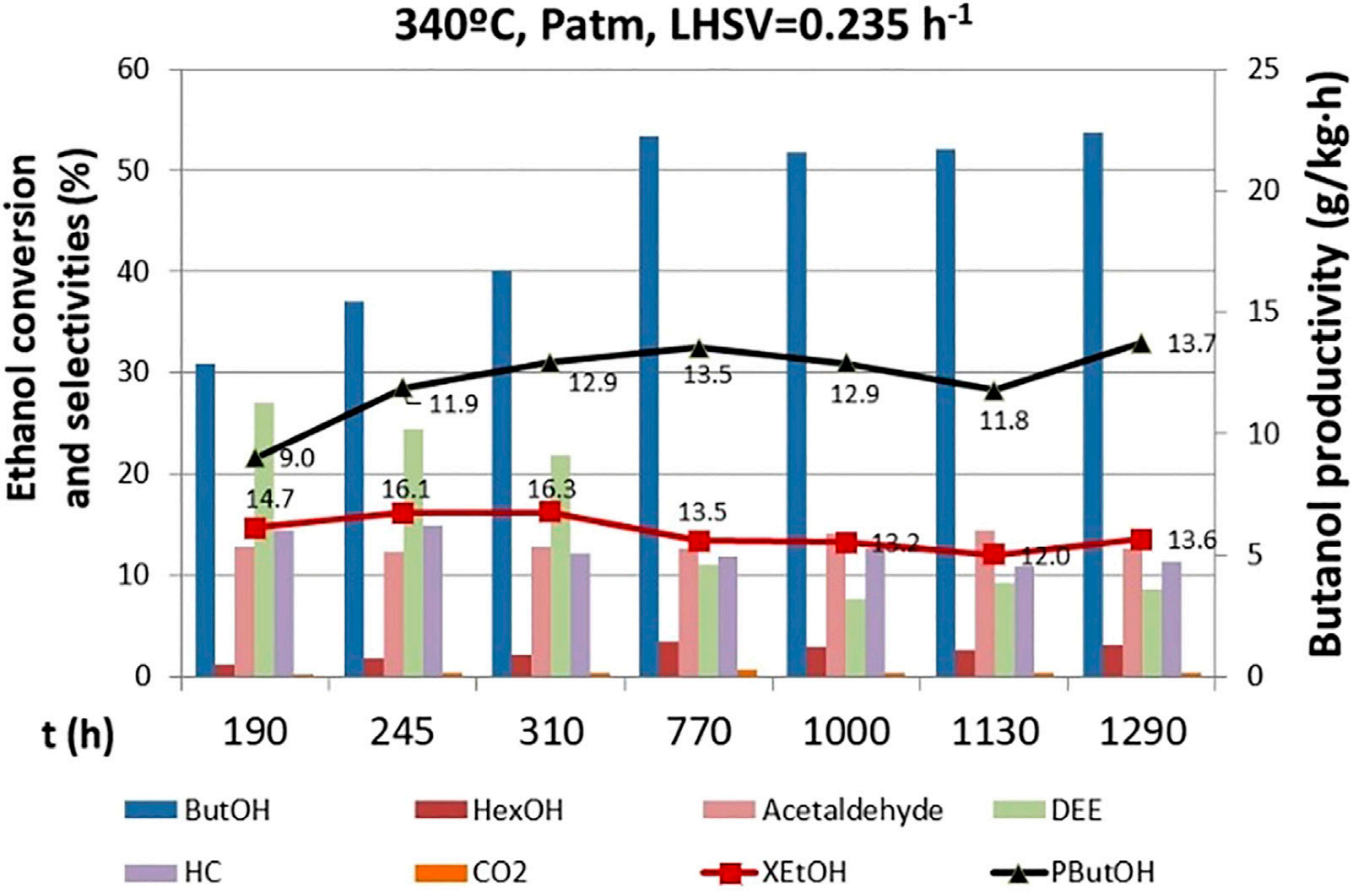
Catalyst stability on time. ButOH = butanol; HexOH = hexanol; HC = hydrocarbons; DEE = diethyl ether.

Catalyst stability was assessed by performing a long-term test of 1,300 h at 340°C, atmospheric pressure (0.39 bar of ethanol partial pressure), and LHSV = 0.235 h^−1^. This long-term test comprised numerous short-term tests performed between the other catalytic tests where the operating conditions were changed. Such a long-term test with over 1,000 h of continuous operation has not yet been reported in literature for this reaction. Indeed, it is important to perform long-term tests since the distribution of products changes over time, but unfortunately, this kind of realistic stability tests are scarce within the academic studies. The catalyst activity along the long-term test did not change significantly, but the product distribution changed from the beginning of the test until 770 h, when it was stabilized (Figure 4). Several different product distributions were observed along time on stream. At the start of the run, low butanol selectivity (∼31%) and high selectivity to diethyl ether (DEE) (∼28%) were observed. DEE is an undesired product, and it is produced from dehydration and coupling of ethanol in acid sites. In this system, Lewis and BrØnsted acid sites are ascribed to alumina. Hence, fine-tunning the presence of this acidic sites by adjusting Mg in the spinel can to guide the selectivity toward the desired products.

DEE selectivity decreased to ∼10% at 770 h of operation, in favor of the n-butanol selectivity (∼52%). This may be because of the catalyst’s structure dynamics during the reaction resulting in a modification of the active centers for dehydration, thus hampering this route as time elapses.

#### Effect of Temperature

**FIGURE 5:**
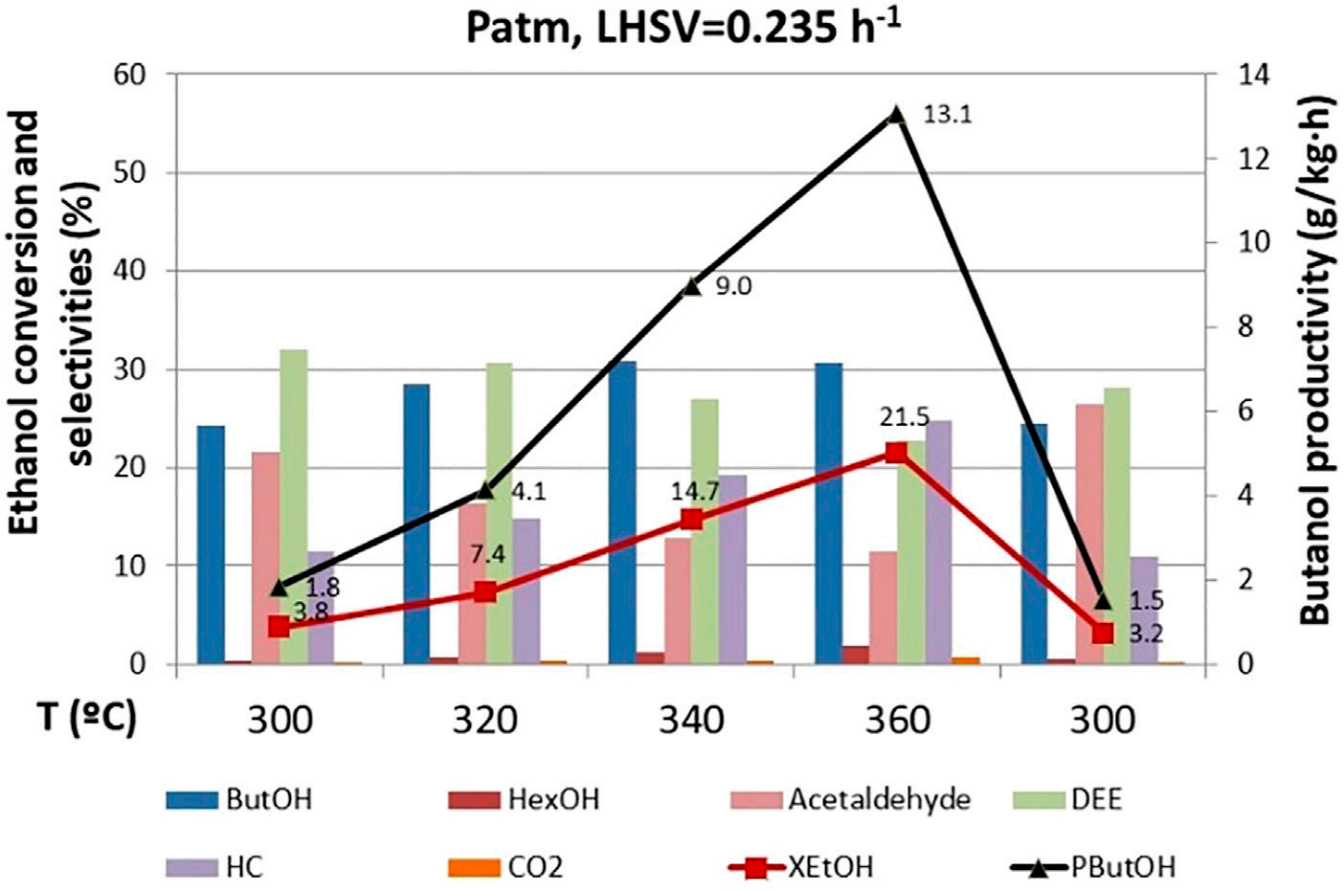
Effect of temperature on ethanol conversion, butanol productivity, and selectivity to products. ButOH = butanol; HexOH = hexanol; HC = hydrocarbons; DEE = diethyl ether.

Temperature has a significant effect on catalyst performance. As expected, ethanol conversion rises with temperature (Figure 5). These tests were carried out before the 770 h operating point, so still high selectivity to DEE was observed. As the temperature increases, the selectivity to DEE decreases, and the selectivity to butanol increases up to a point where it decreases, exhibiting a maximum between 340 and 360°C, since the formation of hydrocarbons (mainly ethene and butenes from the dehydration of the ethanol and butanol, respectively) is more favored at high temperature. Increase of ethanol conversion with temperature prevails over selectivity to butanol, and maximum productivity of butanol is achieved at the highest temperature (360°C). Likewise, the acetaldehyde concentration drops with temperature, evidencing the high reactivity of the aldehydes, which are easily decomposed in the high temperature range. It should be noted that heavy compounds are not formed, with ethene and butenes being the main hydrocarbons observed. Furthermore, for the operation conditions studied, there is a very low CO_2_ production, showcasing the low-carbon footprint in this process when this spinel catalyst is implemented.

#### Effect of Pressure

**FIGURE 6:**
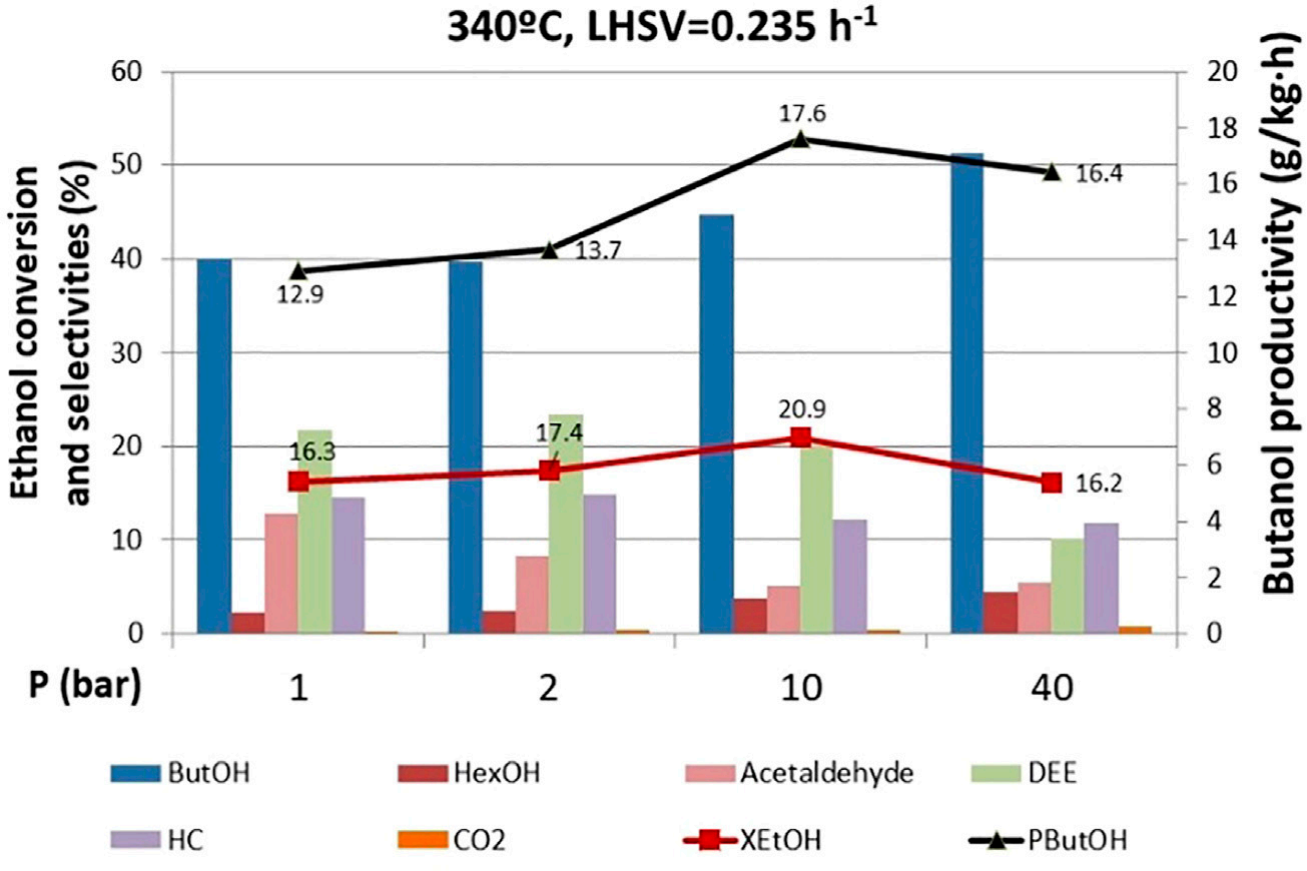
Effect of pressure on ethanol conversion, butanol productivity, and selectivity to products. ButOH = butanol; HexOH = hexanol; HC = hydrocarbons; DEE = diethyl ether.

Pressure effect runs were carried out before the 770 h operating point where DEE was still a major side product (Figure 6). Very interestingly, ethanol conversion is not significantly affected by pressure, which is an additional advantage from the process perspective, since low pressures might suffice to achieve acceptable levels of ethanol conversion, thus saving reaction operating costs. However, the product distribution is remarkably influenced by pressure. At high pressures, the selectivity of butanol reaches a value higher than 50%, while the selectivity to DEE drops to 10%. The latter indicates that the dehydration reaction is unfavored at high pressures, in good agreement with previous results dealing with short-chain alcohol dehydration (Taylor et al., 2010). This is a very relevant observation, since we can adjust the selectivity toward the desired product (i.e., butanol) by adjusting the total pressure in the system without compromising the overall ethanol conversion.

#### Effect of Liquid Hourly Space Velocity

**FIGURE 7:**
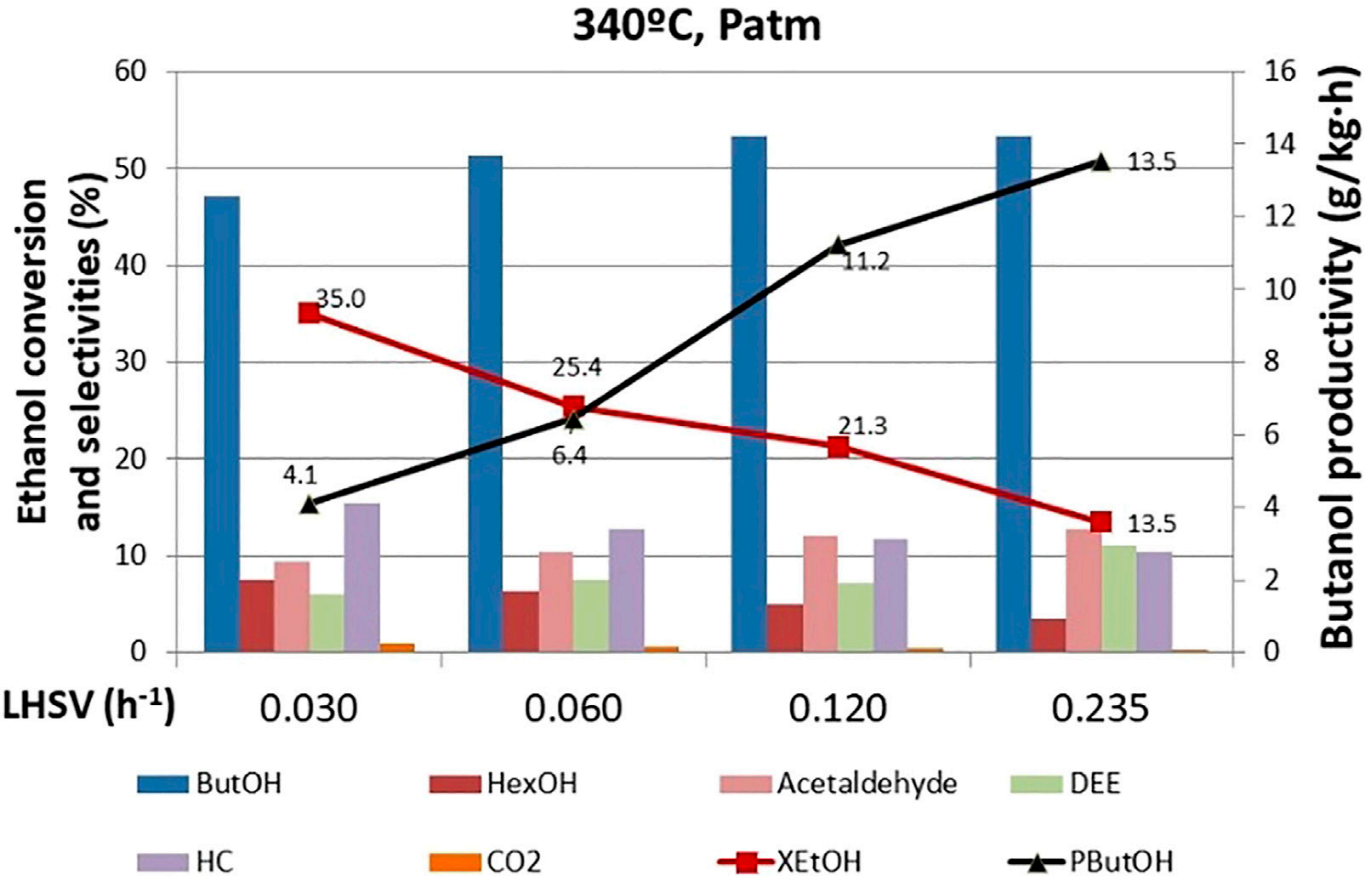
Effect of LHSV on ethanol conversion, butanol productivity, and selectivity to products. ButOH = butanol; HexOH = hexanol; HC = hydrocarbons; DEE = diethyl ether.

The effect of the LHSV on ethanol conversion, selectivity to products, and butanol productivity is shown in Figure 7. These tests were carried out after the 770 h when the DEE selectivity was low. Higher space velocity implies a higher flow rate fed to the reactor per mass of catalyst and, therefore, less contact time between the reactants and the catalyst. Hence, an ethanol conversion drop upon increasing the space velocity was observed. In any case, it must be highlighted that at the lowest studied space velocity (0.03 h^−1^), we reached a highly commendable catalytic performance. The ethanol conversion level hit 35% with 48% selectivity to butanol and 7% selectivity to hexanol, which is the condensation product with higher carbon content. In addition, under these conditions, the selectivity to unwanted products (DEE) is minimized. As far as reaction intermediates is concerned, acetaldehyde is the first intermediate product from ethanol dehydrogenation that eventually evolves to butanol upon a subsequent condensation process as depicted in Supplementary Figure S1. The space velocity screening confirms such mechanistic hypothesis, since at higher space velocity, acetaldehyde concentration within the reaction products increases. Indeed, given the shorter contact time, ethanol conversion to higher carbon content products is hampered, pushing forward the selectivity toward early-stage reaction products such as acetaldehyde. Butanol productivity increases with space velocity because the absolute amount of butanol generated is higher. The effect of increasing the reagent flow prevails over the decrease in ethanol conversion. This is an interesting observation that might help find the optimal reaction conditions. Since the ethanol conversion and the butanol productivity trends are interlaced, we could figure out an intermediate operating point where we reach a suitable trade-off reaction conversion/targeted chemical productivity.

#### Effect of Hydrogen

**FIGURE 8:**
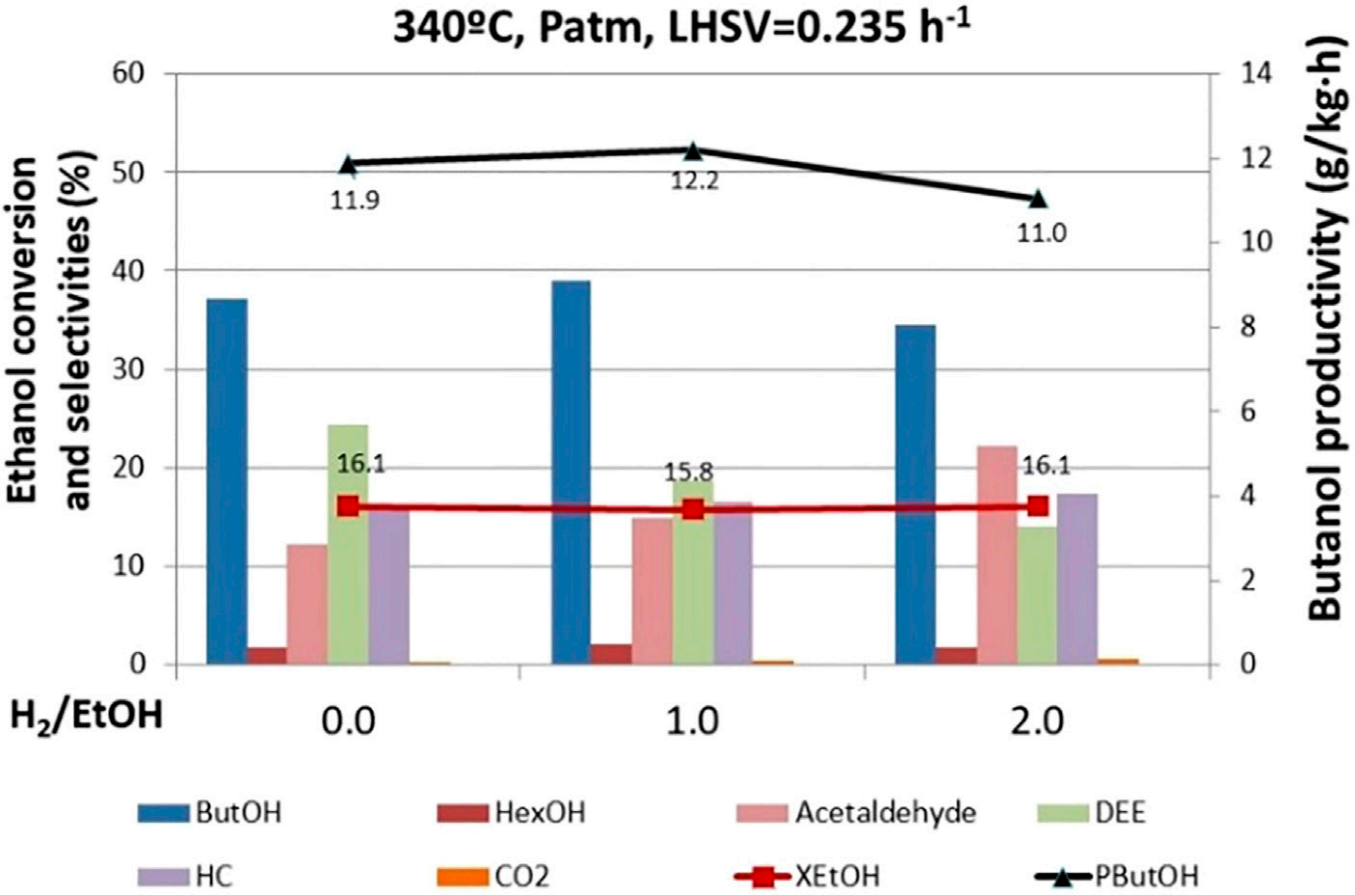
Effect of the ratio H_2_/EtOH on ethanol conversion, butanol productivity, and selectivity to products. ButOH = butanol; HexOH = hexanol; HC = hydrocarbons; DEE = diethyl ether.

According to the Guerbet reaction mechanism, n-butanol is produced by hydrogenation of an intermediate product (crotonaldehyde), yielding hydrogen as a side intermediate product in this route. Thus, if we have in mind a realistic industrial application, a potential improvement could be recycling hydrogen to boost butanol selectivity. Hence, different tests were carried out at 0, 1, and 2 H_2_/ethanol ratios using the mixed oxide spinel catalyst (MgAl_2_O_4_). Ethanol conversion does not change significantly with the H_2_/EtOH molar ratio (Figure 8). However, the H_2_/ethanol ratio affects the end-product distribution. Upon hydrogen co-feeding, the selectivity to butanol increases for H_2_/EtOH ratio 1. Nevertheless, when we co-fed a remarkable excess of hydrogen (i.e., H_2_/EtOH = 2), butanol selectivity decreases to lower values compared to that in the experiment in the absence of H_2_. This is an interesting effect reflecting, on the one hand, the positive impact of small hydrogen concentration as co-feeding reacting. It is very likely that hydrogen is activated on the catalyst surface, becoming more available to react, thus favoring crotonaldehyde hydrogenation. On the other hand, when too much hydrogen is fed, it becomes a competitor for ethanol and the key reaction intermediates partially occupying the active sites and somehow slowing down the reaction progress to the product of interest.

Indeed, Kots et al. (2019) studied the effect of hydrogen on a bifunctional zeolyte catalyst containing palladium and zirconium, reporting a very similar effect. The presence of hydrogen in the gas phase leads to the displacement of ethanol from the metal surface and prevents the formation of surface carbonates and acetates, which are key reaction intermediates to ensure the reaction progress toward advanced products. However, when controlling the hydrogen concentration (for instance, keeping a low H_2_/EtOH ratio), the amount of C4 products increases, suggesting that a controlled dose of hydrogen has a promoting effect on the formation of crotonal as a precursor of butanol, butenes, and butane.

## Conclusion

The effect of operating conditions on the performance of a mixed oxide spinel catalyst (MgAl_2_O_4_) prepared from Mg–Al hydrotalcite was studied in the production of n-butanol from ethanol. Mg–Al spinel catalysts demonstrate highly commendable performance in the Guerbet reaction with a promising activity/butanol selectivity balance and excellent long-term stability. The excellent catalyst stability (for over 1,000 h of continuous operation) is ascribed to the robustness of the Mg–Al spinel phase, which does not suffer from sintering or carbon deposition under the studied conditions, as demonstrated by XRD and TGA. In all the studied conditions, the CO_2_ generation as by-products is minimal, evidencing the low-carbon footprint nature of this route when our catalyst is implemented.

Our results show that the key reaction parameters have a clear impact, which can be fine-tuned to boost the overall performance. For instance, ethanol conversion is promoted upon increasing temperature, while pressure is a key parameter to favor selectivity toward butanol. An adequate trade-off reaction conversion/targeted chemical productivity is possible to fine-tune the space velocity. The overall conversion decreases upon increasing the space velocity, and butanol productivity is boosted at higher space velocities. The impact of hydrogen co-feeding is twofold: relatively small H_2_/EtOH ratios favors butanol selectivity, while high ratios possess H_2_/EtOH competition to get adsorbed and activated, thus slowing the reaction progress toward butanol.

Overall, this study showcases a systematic strategy to optimize the reaction parameters in the Guerbet reaction for bio-butanol production using a suitable spinel catalyst. Upon carefully adjusting, temperature, pressure, space velocity, and reactants co-feeding, promising conversion (35%) and butanol selectivity values (48%) are obtained, opening some room for further investigation in this green route for biofuels/bio-chemicals production.

## Data Availability

The original contributions presented in the study are included in the article/Supplementary Material, and further inquiries can be directed to the corresponding author.
