# Supplementary material for: Insights on Guerbet Reaction: Production of Biobutanol From Bioethanol Over a Mg–Al Spinel Catalyst
Source: Front Chem. 2022 Jul 14;10:945596. doi: 10.3389/fchem.2022.945596 (PMC9329697; doi:10.3389/fchem.2022.945596)
Supplement: Supplementary file 1 [file DataSheet1.PDF]

Supplementary Material

for

Insights on Guerbet reaction: production of biobutanol from bioethanol over a Mg-Al spinel catalyst

S1. Ethanol condensation to n-butanol by Guerbet pathway.
In the generally accepted Guerbet pathway, ethanol is first dehydrogenated to acetaldehyde (R1). The next step involves the formation of 3-hidroxybutanal via aldol condensation of two molecules of acetaldehyde (R2). 3- hidroxybutanal is then dehydrated to crotonaldehyde (R3), which is finally hydrogenated to n-butanol (R4). Globally, two molecules of ethanol are converted to one molecule of n-butanol with the release of one molecule of water [i]. Along with the condensation pathway, several side reactions take place during the process (Figure S1). Such side reactions must be suppressed to increase selectivity to n-butanol.

$$CH_3CH_2OH \rightarrow CH_3CHO + H_2 \qquad \text{(SI-1)}$$

$$2\ CH_3CHO \rightarrow CH_3CHOHCH_2CHO \qquad \text{(SI-2)}$$

$$CH_3CHOHCH_2CHO \rightarrow CH_3CH{=}CHCHO + H_2O \qquad \text{(SI-3)}$$

$$CH_3CH{=}CH_2CHO + 2\ H_2 \rightarrow CH_3CH_2CH_2CH_2OH \qquad \text{(SI-4)}$$

Figure S1. Main and side reactions for ethanol condensation to n-butanol.

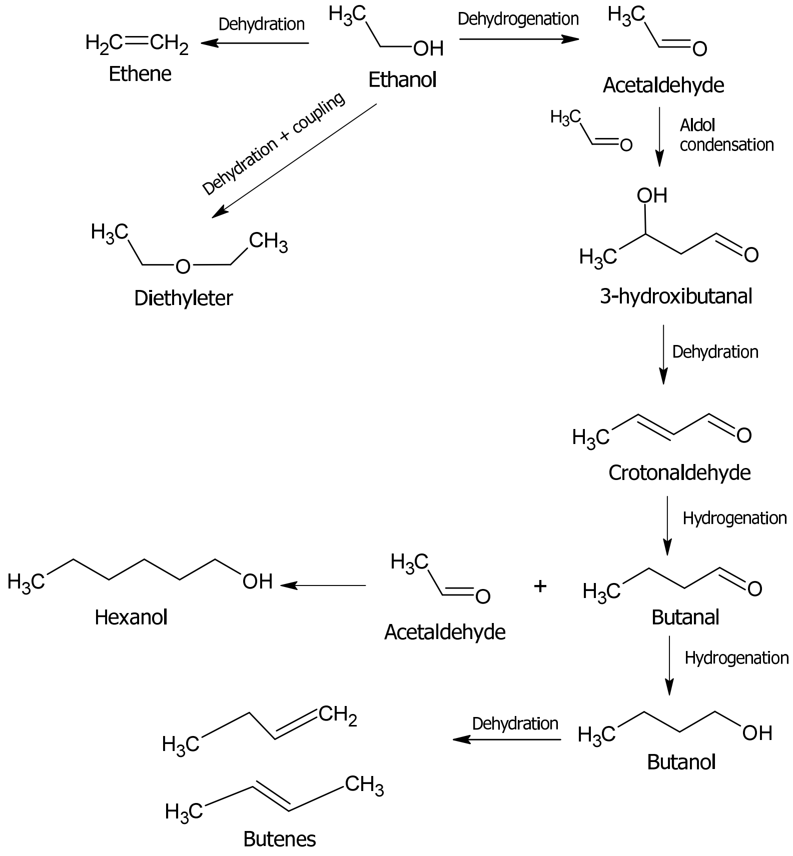

S2. Experimental setup

Figure **S2** shows a diagram of the catalytic reactor experimental setup. Almost pure N_2 and H_2 were supplied from pressurized cylinders and gas flow rates were adjusted by mass flow controllers (MFCs). N_2 was used as inert gases for chromatographic quantification. Liquid ethanol was fed to the reactor by means of a syringe pump. A Bronkhorst mini-coriolis MFC was used to set the ethanol flow rate. The experiments were carried out in a high-pressure fixed bed reactor (inner diameter 8.3 mm, length 330 mm, stainless-steel). A bed of 2 grams of calcined catalyst was placed inside the reactor between two beds of SiC, and all the beds were separated by rock wool. The catalyst was in the shape of granules with a particle diameter in the range of 0.3-0.5 mm and an apparent density of 740 kg/m^3.

An electric oven supplied heat to control the reactor temperature, which was measured by a multipoint type K thermocouple located along the reactor. The pressure downstream of the reactor was controlled with a back-pressure regulator. The reactor outlet stream was analyzed on-line using a GC detection system (Agilent 7890B). The GC is equipped with a double injector, five capillary columns, a thermal conductivity detector (TCD) and two flame ionization detectors (FID) and uses helium as a carrier gas. The system is composed of two channels that work in parallel. The first channel is used to determine H_2, O_2, N_2, CO, CO_2, H_2O, methanol, ethanol and C1-C4 hydrocarbons. To do this, the sample is injected through the valve 1 to a set of 3 capillary columns that allows the separation of these products and they are detected by a TCD. The second channel is used to separate the rest of the products (mainly alcohols and hydrocarbons but also ethers, esters...). Through a deans system, 2 columns and 2 FID detectors, the rest of the products not observed in channel 1 are detected.

All piping from the reactor to the gas chromatograph (GC) was wrapped in heat tracing tape to avoid condensation of reaction products. Downstream the GC, there is a condenser to separate the liquid and gaseous components.

Figure S2. Simplified diagram of the experimental setup.

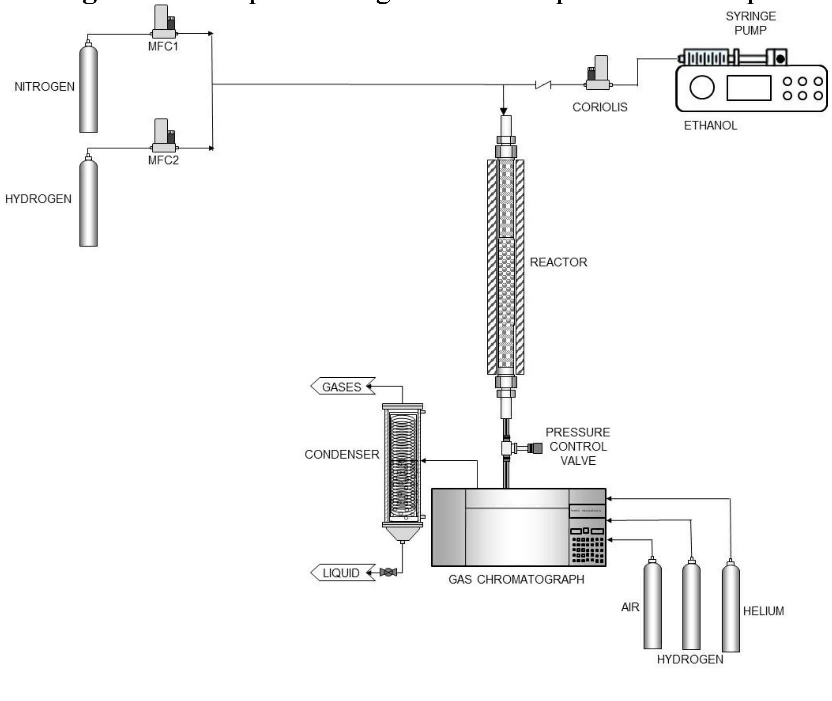

S3. Catalyst textural properties

Figure **S3** (A, B) presents the nitrogen adsorption-desorption and pore size distribution of the calcined catalyst ($MgAl_2O_4$), respectively. According to the IUPAC classification, the solid shows type IV isotherms with H1 hysteresis loop closing, characteristic of mesoporous materials formed by agglomerated or aggregates of spheroidal particles with a uniform pore size [ii]. As can be observed in

Figure **S3** (B), the mesoporous material presents a monomodal pore size distribution with a maximum of ≈18 nm. The $MgAl_2O_4$ sample has a specific surface (S_{BET}) of 83 $m^2\ g^{-1}$ with a pore volume and diameter of 0.5 $cm^3\ g^{-1}$ and 17 nm, respectively. The values obtained are within the standard range of magnesium aluminate spinel calcined at 900 ºC [iii,iv].

Figure S3. (A) N_2 adsorption/desorption isotherm and (B) pore size distribution of calcined catalyst ($MgAl_2O_4$).

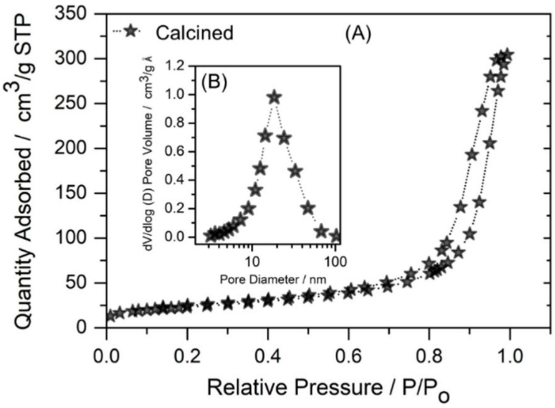

[i] Qi Zhang et al (2016). Towards a green bulk-scale biobutanol from bioethanol upgrading. Journal of Energy Chemistry 25, 907–910.
[ii] G. Leofanti et al (1998). Surface area pore texture of catalysts, Catal. Today, 41, 207-219.
[iii] H. Wang et al (2020). Hierarchical Fe-modified $MgAl_2O_4$ as a Ni-catalyst support for methane dry reforming, Catal. Sci. Technol., 10, 6987-7001.
[iv] S. Sanjabi and A. Obeydavi (2015). Synthesis and characterization of nanocrystalline $MgAl_2O_4$ spinel via modified sol–gel method, J. Alloy Compd., 645, 535-540.
